# Studies on HIV/AIDS Among Students: Bibliometric Analysis

**DOI:** 10.2196/46042

**Published:** 2023-08-04

**Authors:** Na Wang, Runxi Zhang, Zeyan Ye, Guanghua Lan, Qiuying Zhu, Huanhuan Chen, Xiangjun Zhang, Shengkui Tan, Yuhua Ruan, Mei Lin

**Affiliations:** 1 Guangxi Key Laboratory of Major Infectious Disease Prevention Control and Biosafety Emergency Response Guangxi Center for Disease Control and Prevention Nanning China; 2 Guangxi Key Laboratory of Environmental Exposomics and Entire Lifecycle Health School of Public Health Guilin Medical University Guilin China; 3 Department of Clinical Pharmacy and Translational Science College of Pharmacy University of Tennessee Health Science Center Memphis, TN United States

**Keywords:** bibliometric analysis, HIV, acquired immunodeficiency syndrome, AIDS, student, university, college, postsecondary, bibliometric, communicable, sexually transmitted disease, STD, sexual transmission, sexually transmitted infection, STI

## Abstract

**Background:**

In recent years, HIV infection in students has been an ongoing concern worldwide. A large number of articles have been published; however, statistical analysis of the data presented in these publications is lacking.

**Objective:**

This study aimed to detect and analyze emerging trends and collaborative networks in research on HIV/AIDS among students.

**Methods:**

Research publications on HIV/AIDS among students from 1985 to 2022 were collected from the Web of Science Core Collection. A topic search was used for this study, and articles in English were included. CiteSpace was used to generate visual networks of countries/regions, institutions, references, and keywords. Citation analysis was used to discover milestones in the field and trace the roots of the knowledge base. Keyword analysis was used to detect research hotspots and predict future trends.

**Results:**

A total of 2726 publications met the inclusion criteria. Over the past 38 years, the number of publications annually has been on the rise overall. The United States had the highest number of publications (n=1303) and the highest centrality (0.91). The University of California system was the core institution. The main target population of studies on HIV/AIDS among students were medical and university students. These studies focused on students’ knowledge, attitudes, risk behaviors, and education about HIV/AIDS. The recent bursting keywords (gay, sexual health, adherence, barriers, mental health, HIV testing, stigma, and antiretroviral therapy) revealed research trends and public interest on this topic.

**Conclusions:**

This study identified countries/regions and institutions contributing to the research area of HIV/AIDS among students and revealed research hotspots and emerging trends. The field of research on HIV/AIDS among students was growing rapidly. The United States was at the center, and the University of California system was the core institution. However, academic collaboration should be strengthened. Future research may focus on exploring gay students, sexual health, adherence, barriers, mental health, HIV testing, stigma, and antiretroviral therapy.

## Introduction

HIV/AIDS is a chronic infection that affects not only physical health but also social relationships, mental health, quality of life, and economic aspects. Students are the hope of their families and the future of a nation. Approximately 4000 individuals aged 15 years and older become newly infected with HIV every day worldwide, with 27.5% of them aged 15-24 years [1]. In 2017, approximately 19% of individuals aged 15-24 years living with HIV/AIDS in China were students [2]. Students living with HIV/AIDS could be experiencing body image issues; negative feelings; poor self-esteem; and especially at the university level, poor thinking, learning, memory, and concentration [3]. Therefore, the prevention and control of HIV infection in students must receive close attention.

In recent years, HIV infection among students has been an ongoing concern worldwide, such as knowledge of HIV/AIDS, risk behaviors, and HIV prevention education [4]. However, there is no systematic study of global research trends and guidelines in this area. Bibliometric analysis is a branch of quantitative science that has been used as a powerful tool for understanding emerging trends and knowledge structures in research fields and fostering new research ideas [5].

CiteSpace is an essential bibliometric analysis tool that facilitates the detection of emerging trends and mutations in a field [6]. It has been applied to research in more than 60 different scientific fields [7]. It plays an important role in describing keyword co-occurrence and cocited reference networks. CiteSpace can not only predict emerging trends of spatial epidemiology in infectious diseases [8] but also analyze patterns of relationships between nanosciences, health, and biology [9].

There may be articles that use other bibliometric analysis software; however, they only focused on specific students, such as college students [10]. This bibliometric analysis clearly illustrated the milestones and hotspots of research on HIV/AIDS among students from 1985 to 2022. Articles on HIV/AIDS among students were searched using the Web of Science Core Collection (WoSCC). Afterward, CiteSpace was used to perform statistical calculations and generate visual networks to reveal hotspots and frontiers of research.

## Methods

### Data Sources and Search Strategies

**Figure 1:**
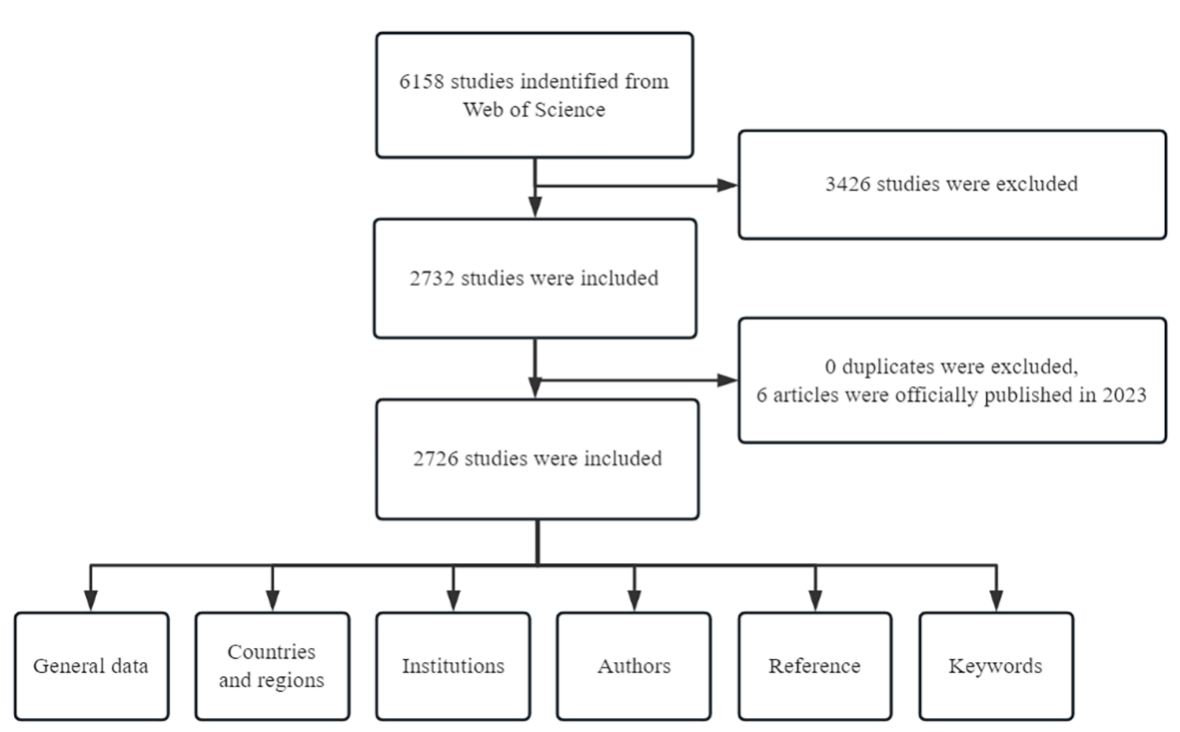
Flowchart of the search strategy and bibliometric analysis process.

The Web of Science database is an authoritative citation information source with the most selective journal coverage [11]. The data search was conducted using WoSCC on March 20, 2023. The research strategies were as follows: *TS=Topic, (TS=“student$”) AND ((TS=“HIV”) OR (TS=“AIDS”) OR (TS=“Acquired Immune Deficiency Syndrome”))*, over the period from 1985 to 2022. A total of 6158 articles were obtained, but 3426 of them were manually excluded for not being relevant to the research content. Table 1 shows the inclusion and exclusion criteria, Figure 1 shows the study flowchart, and Multimedia Appendix 1 shows the complete research strategies and results. Study selection and data extraction were performed independently by 2 authors. Differences of opinion were settled by discussion or referral to a third author.

**Table 1:** Inclusion and exclusion criteria.

	Inclusion criteria	Exclusion criteria
Article type	Article	Reviews, book chapters, editorials, letters, commentaries, meeting abstracts, duplicate literature, etc
Language	English	Spanish, Portuguese, French, German, Russian, etc
Content	HIV/AIDS among students	Aid

### Data Preprocessing

We downloaded all records and references from WoSCC, including authors, titles, journals, years, keywords, abstracts, dates, countries, institutions, and references. Four folders were created, named “Input,” “Output,” “Data,” and “Projects.” All records and references were placed in the “Input” folder and then imported into CiteSpace to remove duplicates. After removing the duplicates, the articles appeared in the “Output” folder by year. Finally, the data in the “Output” folder were copied to the “Data” folder to be ready for analysis by CiteSpace.

### Bibliometric Analysis

CiteSpace (version 6.2.R3) [12] was used to identify countries/regions and institutions contributing to research on HIV/AIDS among students and to reveal research hotspots and emerging trends. CiteSpace parameters were set as follows: (1) time slice from 1985 to 2022; (2) year per slice=1; and (3) pruning=pathfinder or pruning the merged network. Other parameters were set to default values.

Nodes indicated the object of analysis, including countries/regions, institutions, references, and keywords. The more frequently an object appears in the data set, the larger the node. A link between 2 nodes represents a copublishing partnership between 2 countries/regions or institutions [7]. In a network of keyword co-occurrence, a link represents the co-occurrence of 2 keywords in different articles [7]. It implied the association of 2 research contents. The thicker the line, the closer the relationship is between the 2 nodes.

The centrality of a node is a property that quantifies the importance of the node’s position in a network [6]. Betweenness centrality is one of the most commonly used centrality metrics [13]. It measures the percentage of the shortest paths in the network to which a given node belongs [14]. A node with strong betweenness centrality can show a purple ring on the outside [6].

The analysis of keyword bursts can identify hotspots and frontiers that could have an impact on future research [15]. The analysis of citation bursts can reveal articles that had a significant impact in the field [16].

## Results

### General Data

**Figure 2:**
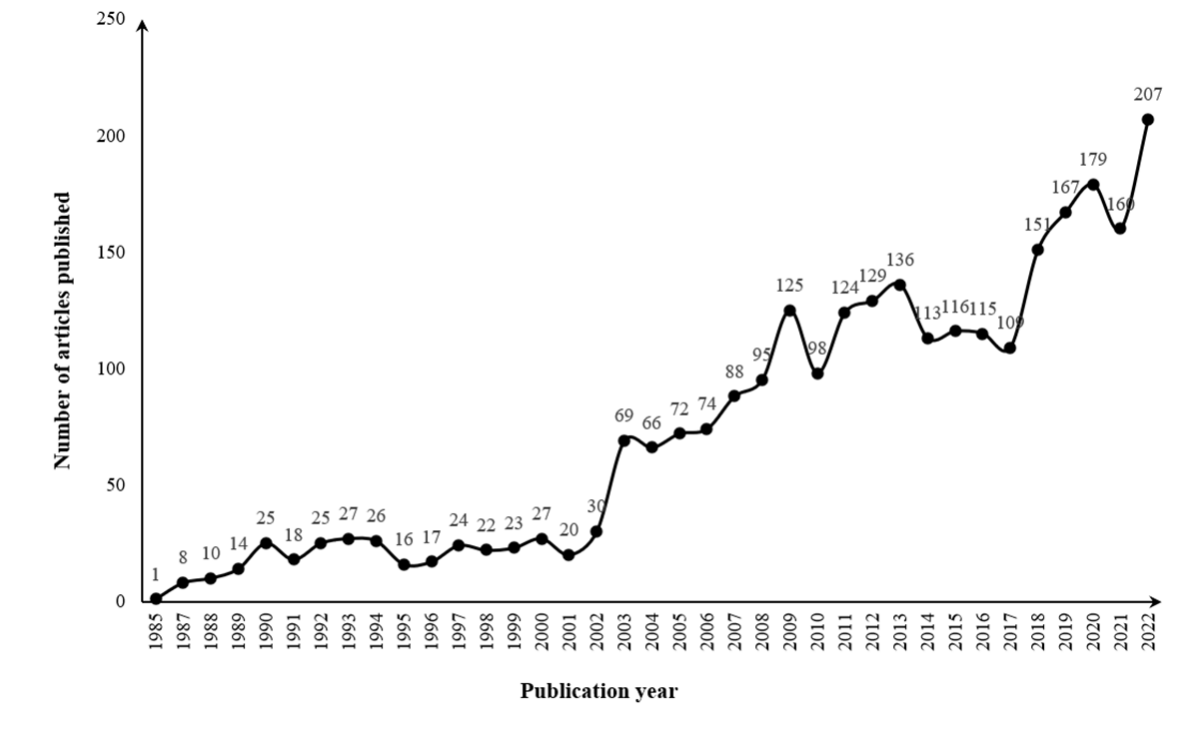
Distribution of articles by publication year, from 1985 to 2022.

A total of 2726 articles were included in this study. The trend in the number of articles reflects the popularity of research and the speed of knowledge growth [17]. The general trend in research on HIV/AIDS among students was on the rise, especially after 2002. The number of articles peaked in 2022, with 207 articles (Figure 2).

Research on HIV/AIDS among students can be divided into 3 stages. The preliminary stage was from 1985 to 2002, with 1 to 30 articles per year. It lasted 18 years but accounted for 12.22% (n=333) of the total number of articles. The research at this time laid the foundation and guided future research. In 1985, Price et al [18] published the first academic article on the assessment of high school students’ perceptions and misperceptions of AIDS. It played an influential and leading role in research on HIV/AIDS among students.

The developmental stage was from 2003 to 2017, with 66 to 136 articles per year. The total number of articles from this time period was 1529 (56.09%). A large number of articles had been accumulated during this stage of research. It can be regarded as a transition between low-level and high-level research. More and more professors and scholars had been attracted to this field, and enthusiasm for this research was high.

The superior stage was from 2018 to 2022, with 151 to 207 articles per year. The total number of articles from this time period was 864 (31.69%). Although there were small fluctuations in this period, studies on HIV/AIDS among students were generally on the rise.

### Research Collaboration

#### Countries/Regions

**Figure 3:**
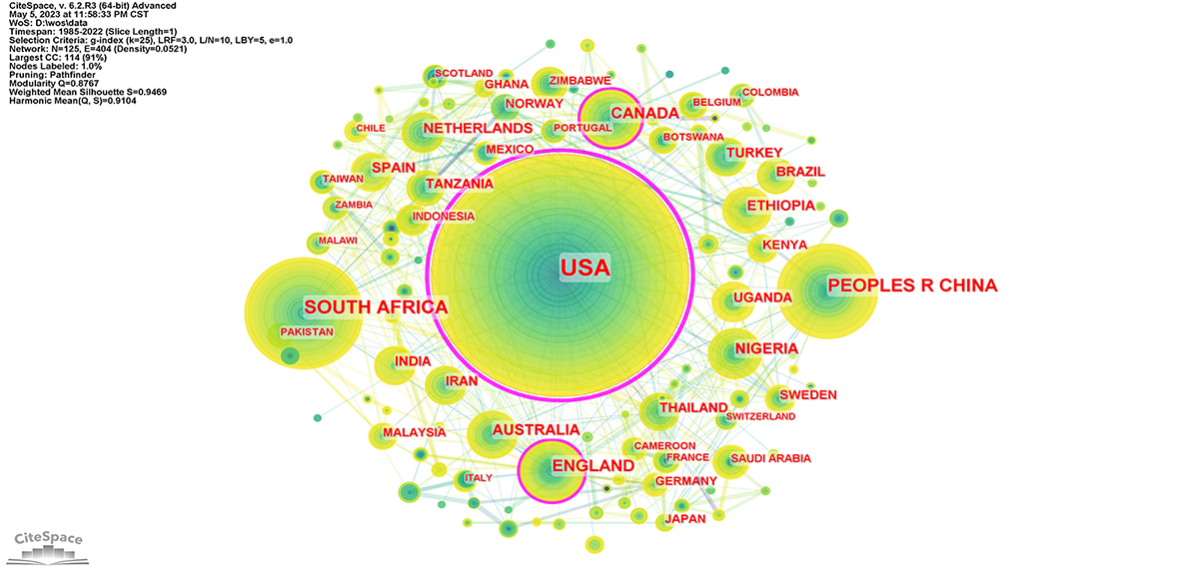
Network of collaborative relationships among countries/regions. CC: co-citations; CST: Central Standard Time; LBY: look back year; L/N: maximum links per node; LRF: link retaining factor; WoS: Web of Science.

An analysis of the geographical distribution of published articles reflects the academic collaboration between countries/regions [19] (Figure 3). The size of the node indicates the number of articles published in different countries/regions [20]. The thicker the link, the closer the cooperation between the countries/regions. The United States contributed the most in terms of the number of articles (n=1303). South Africa (n=295) ranked second, and China (n=209) ranked third. These top 3 countries/regions accounted for 66.29% (1807/2726) of the total number of articles (Multimedia Appendix 2).

The United States was in the lead, with 47.8% (1303/2726) of the total articles and a betweenness centrality of 0.91. It had research collaborations with 69 countries/regions—much more than any other country. The world’s first article on HIV/AIDS among students was published in 1985 by the University of Toledo in the United States [18]. It sparked the beginning of studies on HIV/AIDS among students. Research on HIV/AIDS among students in the United States not only began early but was of high quality.

South Africa had the second-highest number of articles, accounting for 10.82% (295/2726), but had a much lower betweenness centrality (0.09) than the United States. It had research collaborations with 24 countries/regions. The large number of people living with HIV/AIDS in South Africa has attracted the focused attention of experts. Experts have conducted extensive research on students and published numerous articles. In addition, countries/regions heavily affected by HIV/AIDS, such as Nigeria and Ethiopia, had published a large number of articles in this field.

China ranked third with 7.67% (209/2726) of the total articles and a betweenness centrality of 0.07. It cooperated with 19 countries/regions. The first Chinese article on HIV/AIDS among students was published by the University of Hong Kong in 1999 [21]. The proportion of students with HIV in China increased year by year [2]. China’s research in this field started late, but its quality has improved rapidly.

The nodes in the United States, England, and Canada had purple rings, which meant that they had high betweenness centrality. Experts from these countries had extensive international cooperation. In general, the number of articles was higher in countries/regions with high medical standards, such as the United States and England. Some countries/regions with high HIV prevalence also had a large number of articles, such as South Africa, Nigeria, and Ethiopia. However, the top 3 countries/regions for betweenness centrality were all medically advanced. The United States had the most cooperation with other countries/regions. A network of the US-centered academic collaborations has been formed, but collaboration among countries/regions needs to be strengthened.

#### Institutions

**Figure 4:**
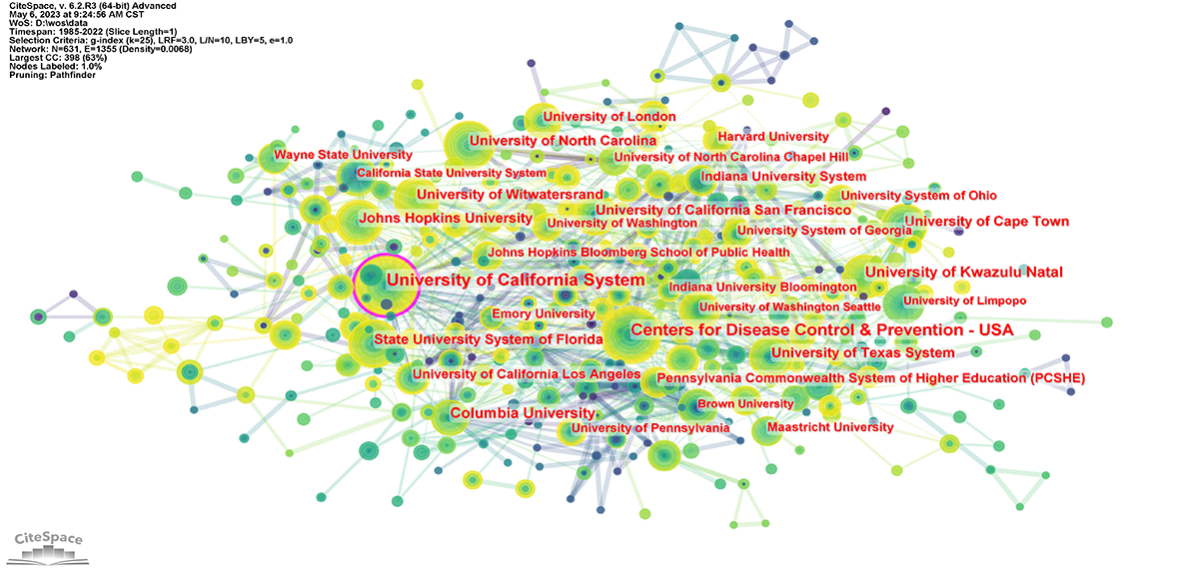
Network of collaborative relationships among institutions. CC: co-citations; CST: Central Standard Time; LBY: look back year; L/N: maximum links per node; LRF: link retaining factor; WoS: Web of Science.

Figure 4 showed the major institutions in research on HIV/AIDS among students. The size of the node indicates the number of articles the institution had published [20]. The thicker the line, the closer the cooperation is between the 2 institutions [20]. Nodes with purple rings have high betweenness centrality.

The University of California system not only had the most publications (n=125) but also had the highest betweenness centrality (0.12). Nine of the top 10 most productive institutions were universities, and the other was the Centers for Disease Control and Prevention. Of these, 8 institutions are from the United States and 2 are from South Africa. Institutions with a high number of publications are mainly concentrated in the United States (Multimedia Appendix 2).

In terms of research collaboration, primary cooperation networks have been formed. The University of California system was the only node with a purple ring, which had the highest betweenness centrality. This implies that its academic influence was so high that it was a central institution in this field. Recently, institutions have been working together more frequently and more closely than ever before.

### Knowledge Base Analysis

Documents cocitation analysis (DCA) refers to the frequency of 2 documents cited in the joint citation list [22]. The network formed by the cocited references can capture the research priorities of the basic science community [23]. Through DCA, we can discover milestones in the field and trace the roots of the knowledge base.

#### Reference Cocitation

**Figure 5:**
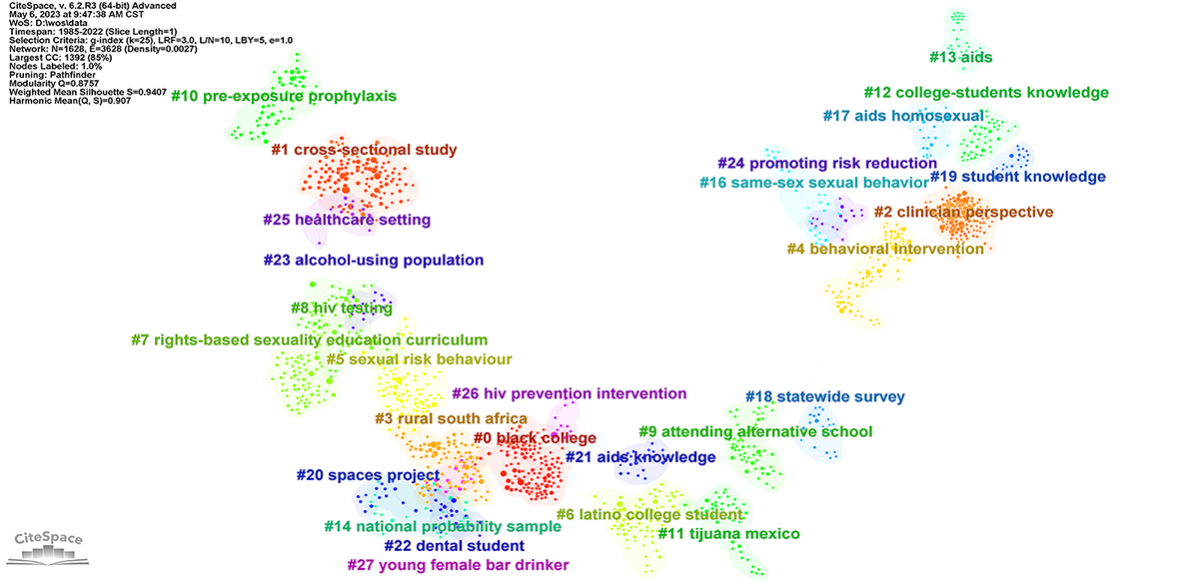
Clusters of reference co-citation. CC: co-citations; CST: Central Standard Time; LBY: look back year; L/N: maximum links per node; LRF: link retaining factor; WoS: Web of Science.

The DCA network consisted of 1628 nodes and 3628 links, and a total of 27 major clusters were formed (Figure 5). Modularity Q can reflect the network structure and the clarity of clustering [24]. It ranges between 0 and 1. The closer the value is to 1, the better the modularity of the network. The silhouette is an indicator of the homogeneity of the members of the entire cluster [24]. It ranges between –1 and 1. The closer the value is to 1, the more homogeneous the cluster members are. In this network, modularity Q was 0.88 and silhouette was 0.94. This suggested that these clusters had analytical significance. CiteSpace provides 3 algorithms to calculate cluster labels: latent semantic indexing, log-likelihood ratio (LLR), and mutual information. Among them, LLR is the best choice to identify the most unique terms to the cluster [12]. Labels extracted by latent semantic indexing tend to capture implicit semantic relationships across data sets, whereas labels selected by LLR and mutual information tend to reflect a unique aspect of a cluster [25]. In the process of clustering, the results obtained by LLR were the most appropriate and most in line with the actual situation. Therefore, the algorithm used in this clustering was LLR.

Clusters #0, #6, #17, #22, #23, and #27 are about the research objects for HIV/AIDS among students. Clusters #3 and #11 are mainly about high-incidence areas of HIV/AIDS among students. Clusters #1, #2, #14, #18, and 20 are related to methods of research on HIV/AIDS among students. Clusters #4, #5, #7, #8, #9, #10, #12, #16, #19, #21, #24, #25, and #26 suggested that the content of the studies was focused on HIV/AIDS knowledge, risk behavior, education, and prevention.

#### Most-Cited Articles

The most-cited article in our data set is Li et al [26] with 24 citations, followed by Hingson et al [27] with 21 citations. Walter and Vaughan [28], Shisana et al [29], and Weinstock et al [30] are tied for third with 19 citations each.

These most-cited articles had much in common. They were almost always related to HIV/AIDS knowledge, attitudes, and behaviors. However, they were studied from different angles. Li et al [26] focused on making suggestions for HIV/AIDS prevention among students from a policy perspective. Walter and Vaughan [28] divided students into an intervention group and a comparison group to evaluate the effect of a HIV curriculum on reducing HIV risk among students. The other 3 articles [27,29,30] analyzed knowledge, attitudes, and behaviors about HIV/AIDS in the form of surveys.

#### Citation Burst

If an article is cited frequently over a short time period, it is considered a reference with strong citation burst [6]. The first article with strong citation burst is Li et al [26] with a burst strength of 12.76. The number of citations to this article increased substantially in 2020 and continues to the present.

#### Sigma

Sigma is a value used in CiteSpace to measure the novelty of a node, which combines the importance of the node in the network structure (betweenness centrality) and the importance of the node in time (bursts) [23]. Nodes with great betweenness centrality and bursts have higher sigma values. The pioneering article by Walter and Vaughan [28] has the highest sigma of 80.96, which means it has both strong betweenness centrality and citation burst.

### Research Hotspots and Evolutionary Trends

#### Analysis of Research Hotspots

**Figure 6:**
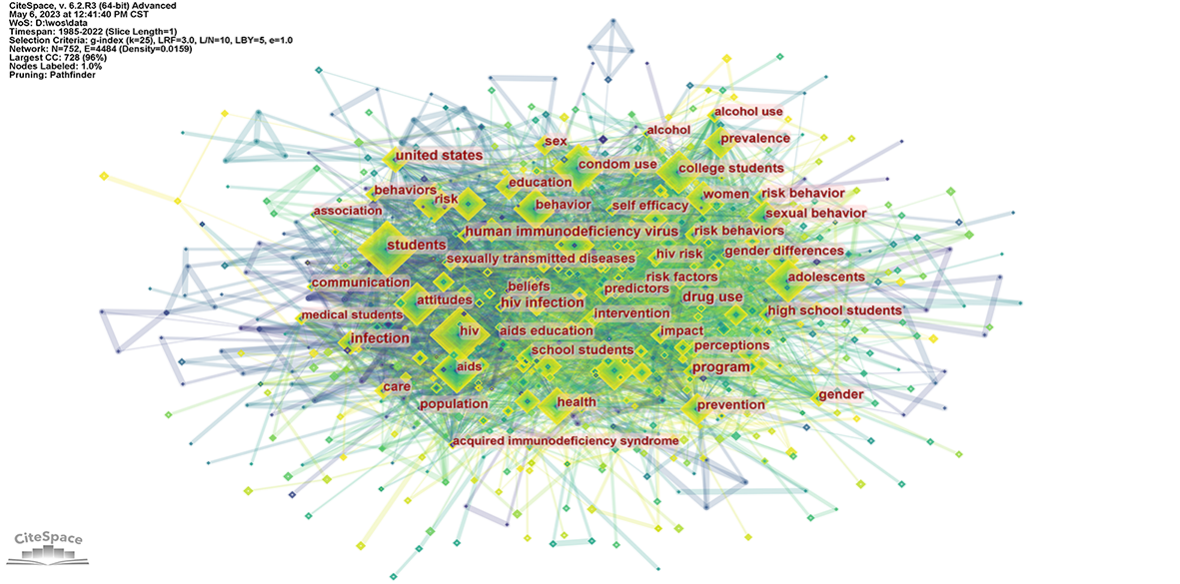
Network of keyword co-occurrence. CC: co-citations; CST: Central Standard Time; LBY: look back year; L/N: maximum links per node; LRF: link retaining factor; WoS: Web of Science.

Keywords are a summary of the content of the article. The analysis of keywords can reveal the development process, explore research hotspots, and especially predict the future development trend of a field [31]. The basic principle of keyword co-occurrence analysis is to calculate the co-occurrence frequency of keywords in different articles and use the co-occurrence frequency to measure the relationship between keywords [8]. There were 752 nodes and 4484 links in this network. Nodes represent keywords. The thicker the line, the more times 2 keywords appear together in different articles (Figure 6).

The keyword with the largest betweenness centrality was “United States” (0.09), whereas the keyword with the greatest count was “HIV” (n=494). These words were mainly related to students’ knowledge, attitude, risk behavior, and prevention of HIV/AIDS. The results implied that the hotspots were closely related to students’ knowledge, attitudes, and risk behaviors toward HIV (Table 2).

**Table 2:** Top 20 high-frequency keywords in studies on HIV/AIDS among students, from 1985 to 2022.

Keyword	Count, n	Centrality
HIV	494	0.04
Students	472	0.05
Attitudes	393	0.03
Condom use	387	0.04
AIDS	386	0.03
Adolescents	369	0.02
Knowledge	346	0.02
Risk	341	0.04
College students	326	0.05
Behavior	286	0.05
HIV/AIDS	276	0.02
Health	239	0.05
Sexual behavior	238	0.05
Prevention	233	0.05
Women	229	0.04
Prevalence	199	0.03
Education	198	0.06
HIV prevention	198	0.03
University students	174	0.02
Infection	172	0.08

#### Keyword Clustering Analysis

**Figure 7:**
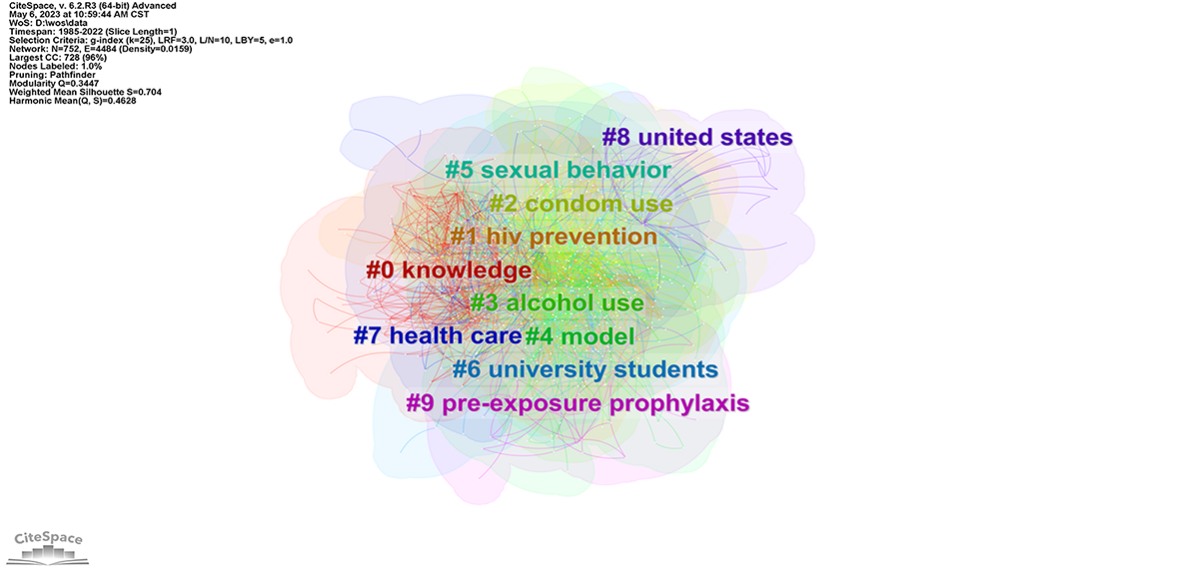
Clusters of keywords. CC: co-citations; CST: Central Standard Time; LBY: look back year; L/N: maximum links per node; LRF: link retaining factor; WoS: Web of Science.

CiteSpace can be used for keyword clustering [15]. Similar keywords can be grouped into a cluster (Figure 7). Normally, modularity Q>0.3 and silhouette>0.7 indicate that map clustering is appropriate [15]. In this network, the modularity Q was 0.34 and the silhouette value was 0.70, implying that the clustering was analytically meaningful. The results obtained by LLR were the most appropriate and most in line with the actual situation. Therefore, this study used the LLR algorithm to extract the clustering labels from the keywords of articles. The results revealed that dental students and university students were the main targets of research on HIV/AIDS among students. The content was focused on knowledge, risk behavior, and prevention (Figure 7 and Table 3).

**Table 3:** Keyword clusters in studies on HIV/AIDS among students, from 1985 and 2022.

Cluster ID	Label (LLR^a^)	Size, n	Silhouette	Major included keywords	Mean years
0	Knowledge	110	0.74	Dental students, attitudes, nursing students, and stigma	2003
1	HIV prevention	103	0.67	Peer education, program, sex education, and South Africa	2005
2	Condom use	96	0.71	Risk, behavior, college students, and HIV infection	1997
3	Alcohol use	90	0.72	Substance use, mental health, sensation seeking, and HIV risk	2005
4	Model	83	0.61	Determinants, health belief model, sex, and planned behavior	2006
5	Sexual behavior	70	0.67	Sexually transmitted infections, risk factors, high school students, and stigma	2007
6	University students	65	0.66	Sexual behavior, risk perception, HIV testing, and sexually transmitted infection	2006
7	Health care	54	0.74	Women, parent-child communication, serious games, and health communication	2012
8	United States	30	0.86	HIV/AIDS, children, sexual minority, and judgments	2002

^a^LLR: log-likelihood ratio.

##### Hotspot Research Objects

In studies of HIV/AIDS among students, the study population consisted mainly of medical students and university students. Medical students have more contact with patients living with HIV/AIDS. Their attitude toward patients living with HIV/AIDS affects the quality of care provided to these patients. They are also at high risk of occupational exposure. Additionally, common university students are at risk of HIV infection through unprotected sex [32].

##### Attitude, Knowledge, and Prevention

Attitudes can help medical students overcome fear and discrimination about HIV/AIDS [33]. For the general university student, HIV-related stigma was one of the strongest barriers to HIV testing and treatment [34]. Through HIV/AIDS education, students gained sufficient knowledge to increase awareness of HIV prevention, reduce the risk of infection, and reduce AIDS-related stigma [35,36].

##### “Risk Behavior” Including Clusters #2, #3, and #5

A cross-sectional study on the risk of HIV transmission among medical students found that 29.13% reported occupational injuries due to needle exposure [37]. Occupational exposure increased the risk of HIV/AIDS infection among medical students. For the common university student, risk behaviors for HIV infection are mainly unprotected sex and substance use [38].

#### Keyword Burst Analysis

An article can be regarded as information flow that arrive continuously over time [39]. The Kleinberg [40] algorithm formalizes the modeling of burst information flow so that burst information flow can be effectively identified. It can be used to detect a sudden increase in research interest in a particular discipline. CiteSpace uses the Kleinberg algorithm to identify emerging research frontiers [6]. It can reveal the frontiers of research at different stages and predict future research directions.

**Figure 8:**
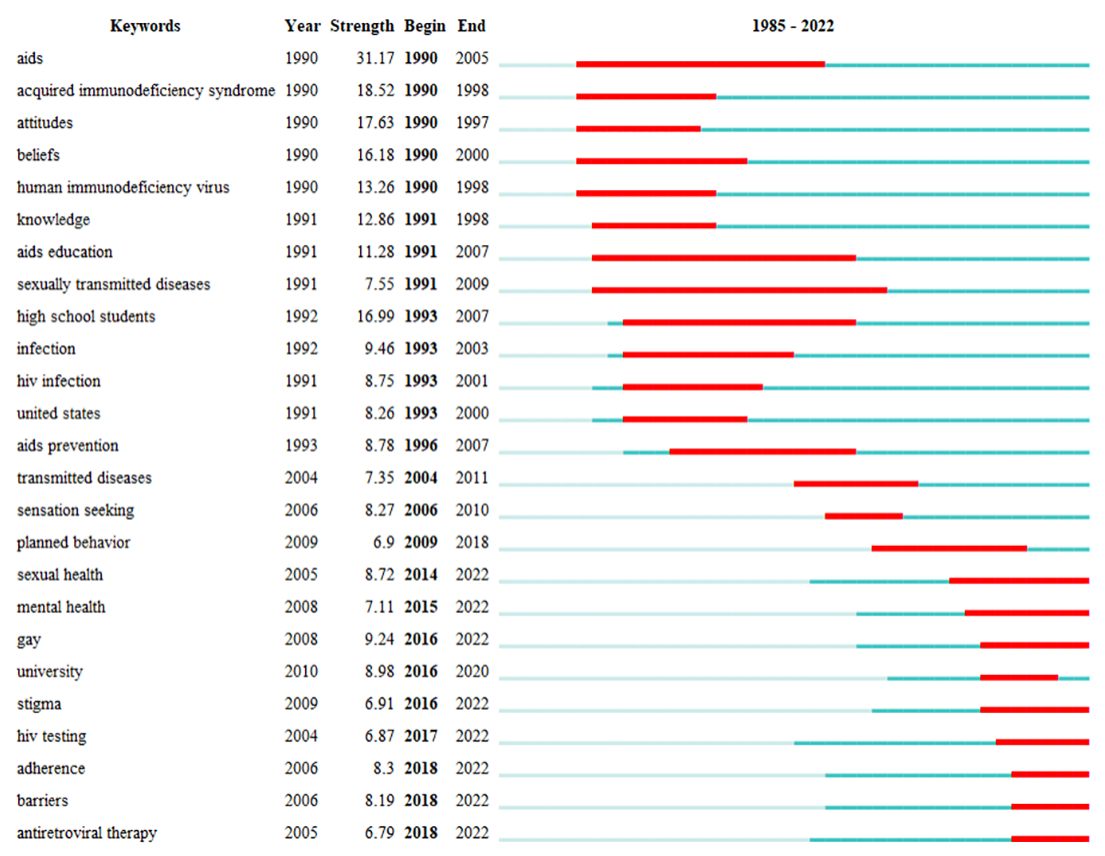
Top 25 keywords with the strongest citation bursts.

Figure 8 shows the top 25 keywords with the strongest citation bursts. “Year” means the year the keyword first appeared. “Begin” refers to the year in which the keyword’s occurrence frequency increased greatly. “End” represents the year in which the popularity of the keyword declined. The blue line represents the timeline, and the red line represents the time when the keyword burst.

From 1985 to 2002, the study population was mainly high school students. Experts focused on students’ knowledge, attitudes, education, and prevention of HIV/AIDS. During this period, there was a wide range of research content and an increase in AIDS research involving students.

From 2003 to 2017, research on HIV/AIDS among students achieved initial results, and the frontiers were deeper than at the previous stage. Compared with the previous period, the object of research was more specific. The hotspot object changed from “high school student” to “gay.” Second, the scope of hotspots had been narrowed. The research frontier had changed from “attitude,” “knowledge,” “education,” and “prevention” to “sensation seeking,” “sexual health,” and “stigma.” Third, the research methods were richer than before. For example, since 2009, the Theory of Planned Behavior has been widely used by experts to estimate students’ attitudes and behaviors toward HIV/AIDS.

From 2018 to 2022, the emerging area of research was adherence, barriers, and antiretroviral therapy. During this period, “adherence” (burst strength=8.30) was the keyword with the strongest citation burst. It is worth noting that several keywords continue to be popular right now, such as gay (9.24), sexual health (8.72), adherence (8.30), barriers (8.19), mental health (7.11), stigma (6.91), HIV testing (6.87), and antiretroviral therapy (6.79).

## Discussion

### Principal Findings

In this study, bibliometric analysis was used to analyze articles related to HIV/AIDS among students from 1985 to 2022. The United States contributed 47.8% of the total articles, and its betweenness centrality (0.91) was much higher than that of other countries/regions. The United States was the core country for studies on HIV/AIDS among students. South Africa had the second-highest number of articles (10.82%), but its betweenness centrality (0.09) was much lower than that of the United States. In general, the number of articles on HIV/AIDS among students was higher in countries/regions with advanced medical systems and some countries/regions with numerous patients living with HIV/AIDS. An academic collaboration network centered on the United States had been formed, but cooperation among countries/regions needs to be strengthened. The cooperation network of institutions was formed. The University of California system is the core institution in this field. In the future, further collaboration among countries/regions or institutions should be encouraged to promote the flourishing of research on HIV/AIDS among students.

With keyword co-occurrence and cluster analysis, the most important topics and information can be easily figured out [16]. If a keyword appears frequently over a short time period, it can be considered a research hotspot [15]. Accordingly, CiteSpace was used to constantly detect changes in high-frequency keywords to accurately explore the trends in the field. The results showed that hotspots in studies on HIV/AIDS among students were constantly changing. In terms of study objects, the early research objects were school students; in the medium term, studies of medical students, college students, and African American students increased; recently, sexual minority communities, especially men who have sex with men (MSM), have been the focus of research. In terms of study content, in the early years, experts studied students’ knowledge and attitudes toward HIV/AIDS and sexual behavior. Then, substance abuse, HIV/AIDS prevention, and education gained a great deal of attention. Recently, research on stigma, HIV testing, and antiretroviral prophylaxis has become increasingly popular. In terms of study methods, earlier studies were conducted mainly through cross-sectional studies and qualitative research. In the medium term, randomized controlled trials were added to the study methods, and the Information-Motivation-Behavioral model was used in research on HIV/AIDS among students. Recently, there has been a tremendous enrichment of research methods. Disorders identification tests, clinical research, implementation science, digital health intervention, and other emerging methods are becoming more widespread in the field. In addition, the application of biopsychosocial model is an emerging practice that has been applied since 2020. The research content was getting deeper and deeper, and the research level kept rising.

The research frontiers have been changing over time since 1985. From 1985 to 2002, the research frontiers were mainly about the initial understanding of HIV/AIDS; from 2003 to 2017, they were mainly focused on the sexual health and stigma of HIV/AIDS; and from 2018 to 2022, they focused on barriers to HIV prevention, HIV testing and treatment adherence, and antiretroviral therapy. Keywords that are still popular today can provide clues for future research, such as gay, sexual health, adherence, barriers, mental health, HIV testing, stigma, and antiretroviral therapy. Risky sexual behavior is popular among college students and has been proven to be a high-risk factor for HIV/AIDS among students [38]. Through strengthening sex health education, students can increase their knowledge of HIV and reduce risky sexual behavior, thereby preventing HIV infection. In addition, the proportion of students who contracted HIV through sexual contact among MSM students is also rising rapidly [41]. However, out of 2726 articles, there were only 343 studies on HIV/AIDS infection in MSM students. It suggests that research on HIV/AIDS among MSM students is still inadequate and should be given more attention. Considering the increasing number of students living with HIV, antiretroviral therapy is particularly important. Experts analyzed the facilitators and barriers to antiretroviral therapy adherence among student with AIDS through qualitative studies [42]. In particular, stigma is a major barrier to students’ adherence to HIV testing and antiretroviral therapy. Reducing stigma can help students improve their adherence to HIV testing and antiretroviral therapy [43]. In the future, research can continue on the hotspots in the suggested direction or try to explore the interaction between the hotspots and find their commonalities to obtain new findings.

In bibliometrics analysis, the citing articles constitute the research frontier, and the cited articles constitute the knowledge base [44]. The most frequently cited articles can be considered milestones in a certain field [45]. Through the analysis of cited articles, it was found that a number of experts used questionnaires to assess students’ knowledge, attitudes, and behaviors regarding HIV/AIDS. Then, problems were identified and summarized to make suggestions for HIV/AIDS prevention and control among students using the information returned. These cited articles laid the foundation for research on HIV/AIDS among students.

### Limitations

There are limitations to this study. First, our study focused on English publications, which inevitably missed some important studies published in other languages. Second, although we used WoSCC in our bibliometric analysis, there may be some articles from other databases (eg, Scopus, MEDLINE, and PubMed) that were not retrieved. However, this study provides a bibliometric analysis of publications on HIV/AIDS among students and is based only on Web of Science data. Through visual analysis software, readers can clearly understand the number of articles, academic cooperation, research hotspots, and research frontiers. It provides hotspots and emerging trends for future research.

### Conclusions

The study identified countries/regions and institutions contributing to the research area of HIV/AIDS among students and revealed research hotspots and emerging trends. The field of research on HIV/AIDS among students was growing rapidly. The United States was at the center, and the University of California system was the core institution. However, academic collaboration should be strengthened. Future research may focus on exploring gay students, sexual health, adherence, barriers, mental health, HIV testing, stigma, and antiretroviral therapy.

## Appendix

Multimedia Appendix 1 Research strategies and results.

Multimedia Appendix 2 Countries/regions and institutions data.

## Abbreviations

DCA: documents cocitation analysis
LLR: log-likelihood ratio
MSM: men who have sex with men
WoSCC: Web of Science Core Collection

## Data Availability

All data generated or analyzed during this study are included in this published article and its Multimedia Appendices.
