# Supplementary figures and images for: Studies on HIV/AIDS Among Students: Bibliometric Analysis
Source: Interact J Med Res. 2023 Aug 4;12:e46042. doi: 10.2196/46042 (PMC10439465; doi:10.2196/46042)

# Researching strategies and results


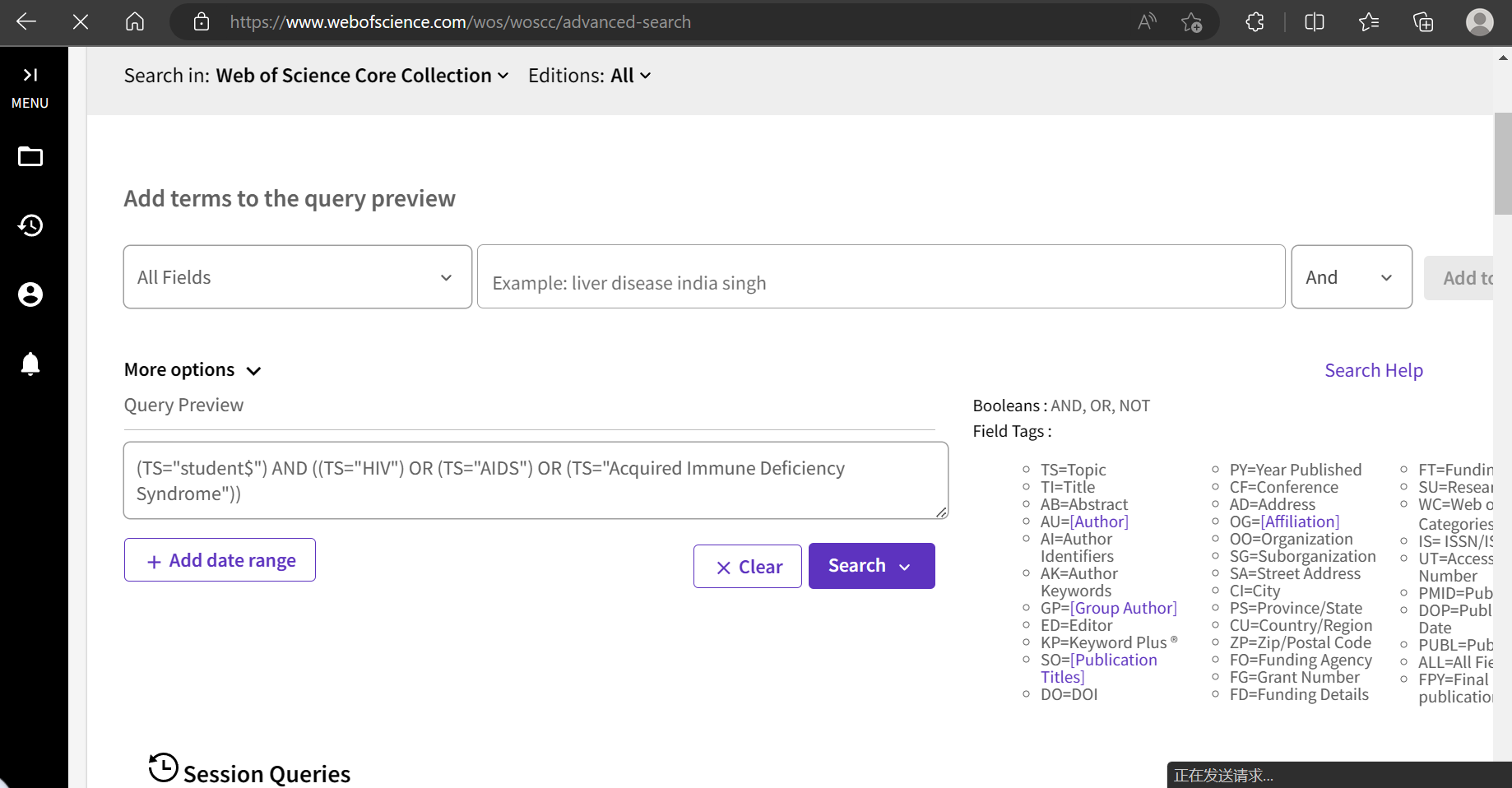


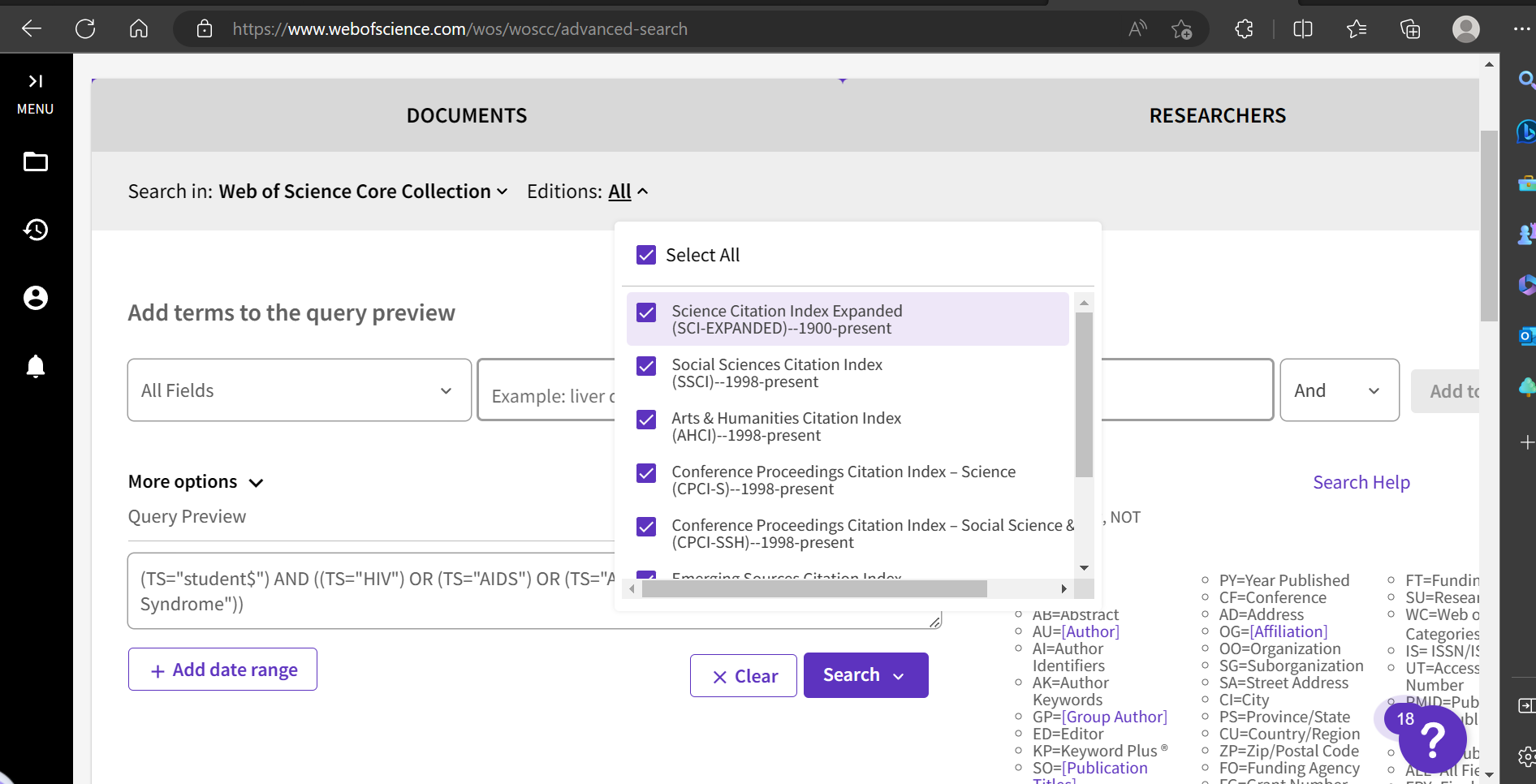


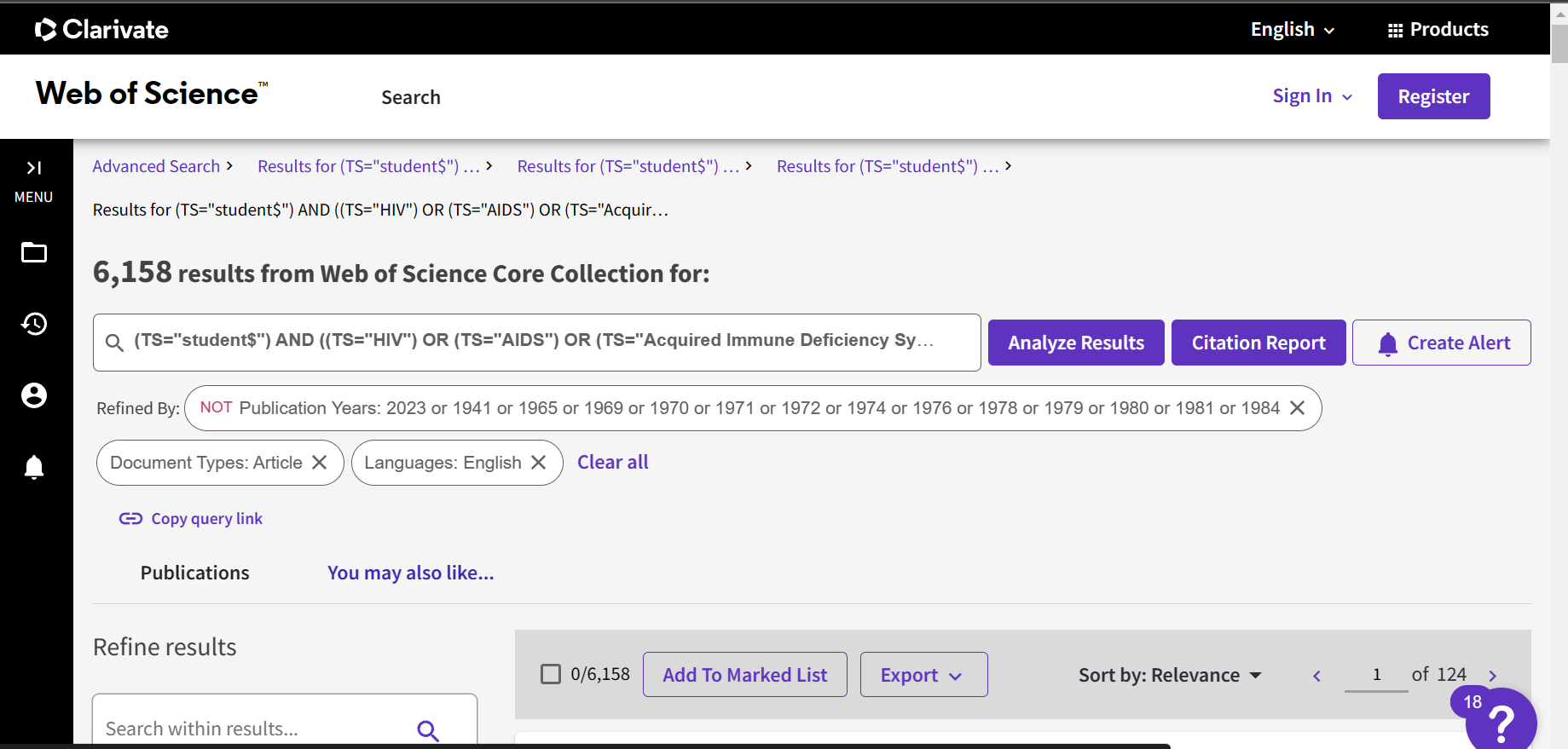

Supplement: Multimedia Appendix 1 [file ijmr_v12i1e46042_app1.zip › Multimedia Appendix 1/Researching strategies and results.docx]
