# Supplementary material for: Studies on HIV/AIDS Among Students: Bibliometric Analysis
Source: Interact J Med Res. 2023 Aug 4;12:e46042. doi: 10.2196/46042 (PMC10439465; doi:10.2196/46042)
Supplement: Multimedia Appendix 2 [file ijmr_v12i1e46042_app2.docx]

# Countries and institutions data results

Table S1. Top 10 regions/countries in studies on HIV/AIDS among students, 1985 - 2022.

| Rank | Count | Centrality | Year | Country |
| --- | --- | --- | --- | --- |
| 1 | 1303 | 0.91 | 1987 | USA |
| 2 | 295 | 0.09 | 1990 | SOUTH AFRICA |
| 3 | 209 | 0.07 | 1999 | PEOPLES R CHINA |
| 4 | 118 | 0.18 | 1988 | ENGLAND |
| 5 | 115 | 0.11 | 1990 | CANADA |
| 6 | 78 | 0.07 | 1989 | AUSTRALIA |
| 7 | 75 | 0.02 | 2001 | NIGERIA |
| 8 | 60 | 0 | 2004 | ETHIOPIA |
| 9 | 57 | 0.06 | 1999 | SPAIN |
| 9 | 57 | 0.05 | 1991 | NETHERLANDS |

Table S2. Top 10 Institutions in studies on HIV/AIDS among students, 1985 - 2022.

| Rank | Count | Centrality | Year | Research Institute |
| --- | --- | --- | --- | --- |
| 1 | 125 | 0.12 | 1987 | University of California System |
| 2 | 91 | 0.06 | 1990 | Centers for Disease Control & Prevention - USA |
| 3 | 57 | 0.02 | 1992 | University of Kwazulu Natal |
| 3 | 57 | 0.07 | 1989 | Columbia University |
| 5 | 53 | 0.04 | 2005 | State University System of Florida |
| 6 | 52 | 0.03 | 1989 | University of North Carolina |
| 7 | 51 | 0.06 | 1990 | University of California San Francisco |
| 8 | 49 | 0.04 | 1999 | Johns Hopkins University |
| 9 | 45 | 0.05 | 1993 | University of Cape Town |
| 10 | 44 | 0.02 | 2000 | University of Texas System |
